# Post-bariatric surgery lab tests: are they excessive and redundant?

**DOI:** 10.1007/s00464-019-07216-9

**Published:** 2019-11-01

**Authors:** Terri Menser, Jose Muniz Castro, Adriana Lopez, Stephen L. Jones, Bita A. Kash, Vadim Sherman, Nabil Tariq

**Affiliations:** 1 Department of Surgery, Houston Methodist, 6550 Fannin Street, Smith Tower, Suite 1601, Houston, TX 77030 USA; 2 Department of Surgery, Weill Cornell Medical College, New York, NY USA; 3 Center for Outcomes Research and Department of Surgery, Houston Methodist, Houston, TX USA; 4 Department of Healthcare Policy and Research, Weill Cornell Medical College, New York, NY USA

**Keywords:** Bariatric surgery, Postoperative period, Clinical laboratory tests, Nutrition assessment, Unnecessary procedures, Outcomes research

## Abstract

**Introduction:**

Following bariatric surgery, ongoing postoperative testing is required to measure nutritional deficiencies; the purpose of this study was to quantify the prevalence of these nutritional deficiencies based on two-year follow-up tests at recommended time points.

**Methods and procedures:**

A retrospective data analysis was conducted of all laboratory tests for bariatric patients who underwent surgery between May 2016 and January 2018 with available lab data (*n *= 397). Results for nine different nutritional labs were categorized into six recommended postoperative time periods based on time elapsed since the procedure date. Binary variables were created for each laboratory result to calculate descriptive statistics of abnormalities for each lab test over time and used in the individual GEE logistic regression models. Grouped logistic regression examined the total nutritional deficiencies of the nine combined nutrients considering total available labs.

**Results:**

Multiple lab tests indicated a very low frequency of abnormalities (e.g., Vitamin A, Vitamin B12, Copper, and Folate). Many of the nine included nutritional labs had an average deficiency of less than 10% across all time points. The grouped logistic model found preoperative nutritional deficiency to be predictive of postoperative nutritional deficiency (OR 3.70, *p* < 0.001).

**Conclusions:**

We found the vast majority of routine lab test results to be normal at multiple time points. Current practice can add up to significant lab expenses over time. The frequency of postoperative testing in this population may be redundant and of very little value. Unnecessary follow-up laboratory testing costs the patients and the health care system in both time and resources. Patients with preoperative deficiencies appear to be at higher risk for nutritional deficiencies when compared to bariatric surgery patients that did not have preoperative nutritional deficiencies. Future research should focus on defining cost effective postoperative lab testing guidelines for at risk bariatric patients.

The American Society for Metabolic & Bariatric Surgery (ASMBS) guidelines for postsurgical tests following bariatric procedures in the USA have remained unchanged for the last decade. The guidelines call for frequent and comprehensive labs that can equate to significant costs. While bariatric surgical procedures are able to effectively reduce the amount of caloric consumption through anatomic and physiologic alteration, nutritional deficiency can be an additional unintended result [1, 2]. To remedy the latter, patients are prescribed nutritional supplements and undergo a series of follow-up blood tests following bariatric surgery to detect potential nutritional deficiencies. It is also established that “Low vitamin concentrations can be caused by dietary and lifestyle habits, abnormal body composition, systematic inflammation, and chronic disease” [3], so detailed plans are available to guide vitamin and mineral supplementation post bariatric surgery [4], and regular blood testing for nutritional deficiencies is the standard of care.

The ASMBS guidelines recommend postoperative blood testing every 3 months for the first year, every 6 months for the second year, and annually thereafter regardless of the type of bariatric procedure performed [5]. Despite procedural changes in bariatric procedures in recent decades [6], and other bariatric guidelines that recommend less frequent postoperative lab testing [7], the recommended testing frequencies have not changed in the USA. With 228,000 bariatric surgeries occurring annually in the USA, and with bariatric follow-up laboratory tests estimated to be roughly $1600 [8], the value of frequent postoperative laboratory testing relative to cost is an appropriate question. A systematic review of micronutrient deficiencies following bariatric surgery found inconsistent results in the percentage deficit by nutritional lab [9]. A recent study examined an algorithm that identified less frequent moderate deficiencies post bariatric surgery; the use of the algorithm was estimated to have a minimum cost saving of 14% [10].

Studies examining the appropriateness and effectiveness of specific laboratory tests for a defined population are increasingly common in the era of both patient-centered care and population health. The purpose of our study was to better understand the rate of nutritional deficiencies post bariatric surgery as the initial step in evaluating the current postsurgical lab guidelines. It is also important to examine how postsurgical nutritional status may differ for patients by bariatric procedure type.

## Materials and methods

All available postoperative labs were collected for retrospective analysis for patients who underwent bariatric surgery from May 2016 to December 2018 (*n *= 397). The crude estimation of the prevalence of out-of-range clinical labs and deficiencies for nutritional labs were calculated using all available lab results for nine different nutritional labs. The postoperative lab data were analyzed at increments recommended by ASMBS (i.e., 3, 6, 9, 12, 18, and 24 months). When more than one lab was available for a defined time period, the lab closest to the respective time point was included in this analysis. Binary variables were created for each laboratory result indicating out-of-range or deficient nutrition followed by calculation of descriptive statistics for each lab test for each recommended time period. This study was approved by the Houston Methodist Research Institute IRB (Protocol ID: Pro0018223) with a waiver of consent for minimal risk research.

### Statistical analyses

Individual GEE logistic regression models for each individual nutrient were used (accounting for repeated measures) in addition to a grouped logistic regression model to test the hypothesis that preoperative nutritional deficiencies is predictive of deficiencies postoperatively. For grouped data, blogit was utilized and is defined as “the maximum likelihood estimator (the same as logit or logistic) but…rather than having individual observations, [the] data are organized so that each observation records the number of observed [met and unmet outcomes]” [11]). The data used in this study are longitudinal (i.e., the initial preoperative measures plus up to six postoperative data points, covering more than two calendar years). It is appropriate to look at this data as blocked/grouped data, giving equal weight to each measure of the occurrence of nutritional deficiencies because it accounts for differences in the denominator (i.e., the number of labs completed). All analyses were completed using using Stata 14 (StataCorp LLC, College Station, TX, USA).

### Dependent variable

We identified nine nutritional labs of interest (copper, ferritin, folate, iron binding capacity, Vitamin A, Vitamin B1, Vitamin B12, Vitamin D, and zinc) that could have up to six values for the two years following bariatric surgery. These nine dependent variables were binary, coded as 1 indicating nutritional deficiency, because it is assumed that the probability of an event occurring is *P*(*Y *= 1). Grouped logistic regression takes into account the count of the total results from a varying number of attempts. Therefore, the dependent variable (DV) numerator was the summed count of labs that were outside the defined threshold, respective to each of the nine lab tests, and the DV denominator was the count of total lab results across all nine labs. The same data without summation were used in the individual GEE logistic regression models.

### Independent variables

#### Preoperative nutritional deficiencies

Given the relationship between preoperative nutritional deficiencies and post-bariatric occurrence of nutritional deficiencies [12], our main independent variable of interest was a binary measure of preoperative lab deficiency. For the grouped logit model, we defined this variable as deficient if one or more of the nine preoperative lab results were deficient/outside the set threshold. If there were no postoperative lab results available for any individual nutrient, the preoperative value was redefined as ‘missing’ to exclude preoperative deficiencies that did not have a postoperative match. A count of deficiencies across the nine nutrition variables was created and used to create the binary preoperative deficiency, defined as one or more preoperative nutritional deficiencies.

#### Comorbidities and medical history

Each of the 17 conditions defined by the Charlson Index [13] were examined for potential model inclusion through univariate analyses; these variables were created at patient level using specified ICD-10 codes. Renal failure and diabetes were included in the model. The Metabolic and Bariatric Surgery Accreditation and Quality Improvement Program (MBSAQIP) offered additional comorbidities, specifically hypertension (defined by the use of hypertension medications), hyperlipidemia, and previous surgery.

## Results

We examined 9 different lab values at 6 different postoperative time periods to describe the prevalence of out-of-range clinical labs and deficiency for nutritional labs (see Table 1). These labs were nutritionally related, which guided the decision to focus on nutritional deficiencies in the grouped logistic regression. It is notable that Vitamin A was deficient in 1.77%, Vitamin B12 was deficient in 1.05%, and copper in 1.51%.

**Table 1:** Total average nutritional deficiencies across six time points

Lab test	Deficiency threshold	3 Months (%)	6 Months (%)	9 Months (%)	12 Months (%)	18 Months (%)	24 Months (%)	Average (%)
Vitamin D	< 30	30.34	33.20	50.00	28.00	31.82	46.15	36.59
Ferritin level	< 30	18.46	22.67	26.47	30.65	31.25	20.00	24.92
Zinc	< 60	5.91	11.06	10.00	7.08	11.11	0.00	7.53
Iron binding	≥ 400	3.95	7.44	4.35	17.53	17.95	8.33	9.93
Folate	< 4.8	3.70	4.50	4.76	4.95	0.00	0.00	2.99
Vitamin B1	< 70	14.09	9.09	25.00	8.65	5.00	0.00	10.31
Vitamin A	< 0.3	5.09	3.32	0.00	2.20	0.00	0.00	1.77
Copper	< 70	0.42	0.44	4.76	0.87	2.56	0.00	1.51
Vitamin B12	< 211	0.75	1.62	3.13	0.80	0.00	0.00	1.05

Patients who underwent bariatric surgery revisions were not included in the analysis in order to be able to compare only primary sleeve gastrectomies and Roux-en-Y procedures. Additionally, if included demographic information were unavailable, those patients were omitted (*n *= 61), resulting in a final sample of 397. The patient characteristics from this final sample are displayed in Table 2.

**Table 2:** Baseline characteristics

Characteristic	All	Sleeve gastrectomy	Roux-en-Y
Total, *N* (%)	397 (100)	172 (43.32)	225 (56.68)
Females, *N* (%)	333 (83.88)	142 (82.56)	191 (84.89)
Age, years, mean	48.12	46.58	49.31
Race, White, *N* (%)	250 (62.97)	90 (52.33)	160 (71.11)
BMI, pre-surgery, mean	44.38	45.61	43.43
Comorbidities			
Hypertension, *N* (%)	224 (56.42)	103 (59.88)	121 (53.78)
Diabetes mellitus, *N* (%)	125 (31.49)	40 (23.26)	85 (37.78)
Renal disease, *N* (%)	42 (10.58)	22 (12.79)	20 (8.89)
Hyperlipidemia, *N* (%)	109 (27.46)	40 (23.26)	69 (30.67)
Previous surgery, *N* (%)	96 (24.18)	15 (8.72)	81 (36.00)

In an effort to understand how patient characteristics, comorbidities, medical history, and procedure type may affect postoperative nutritional deficiencies, the grouped logit model was used (Table 3) as this model allowed for adding additional covariates. Preoperative nutritional deficiency is associated with an increased risk of nutritional deficiencies being observed in the post-procedure period (OR 3.70, *p* < 0.001). There is a statistically significant protective association for patients with hyperlipidemia (OR 0.70, *p* < 0.006). Other factors that were found to be significantly associated with postoperative nutritional deficiencies include age, renal disease, and previous surgery.

**Table 3:** Grouped logistic regression examining the nutritional deficiencies post bariatric surgery

Variable	Odds ratio (95% CI)	*p* value
Preoperative nutritional deficiency (≥ 1)	3.70 (2.69, 5.10)	< 0.001*
Age		
18–40	(reference)	
41–50	0.63 (0.50, 0.78)	< 0.001*
51–65	0.55 (0.43, 0.69)	< 0.001*
65+	0.62 (0.40, 0.94)	0.026*
Gender		
Male	(reference)	
Female	1.34 (1.01, 1.76)	0.041*
Race		
Caucasian	(reference)	
Black	1.02 (0.83, 1.26)	0.821
Other	0.91 (0.51, 1.61)	0.736
Ethnicity		
non-Hispanic	(reference)	
Hispanic	1.05 (0.82, 1.35)	0.678
Body Mass Index		
< 30	(reference)	
30–50	1.26 (0.62, 2.56)	0.514
> 50	1.07 (0.52, 2.22)	0.851
Bariatric procedure type		
Sleeve gastrectomy	(reference)	
Roux-en-Y	1.15 (0.94, 1.40)	0.176
Previous surgery	1.40 (1.13, 1.73)	0.002*
Diabetes	0.99 (0.80, 1.24)	0.944
Renal disease	1.83 (1.39, 2.42)	< 0.001*
Hypertension	0.92 (0.76, 1.12)	0.417
Hyperlipidemia	0.70 (0.54, 0.90)	0.006*

*Statistically significant

Assessing the nutrients individually allowed examination of how preoperative deficiency was related to postoperative deficiency for that same nutrient. We did include time to gain a sense of which of the recommended testing points were statistically significant in our patient population. However, we were not able to estimate a model for either copper or Vitamin A given the lack of variability in deficiency status for these nutrients. A summary of the results of the seven GEE models are displayed in Table 4. In each of the models, preoperative nutritional deficiency predicts postoperative deficiency at a statistically significant level with odds ratios ranging from 2.78 to 29.76. Procedure type was included in these models to understand if the surgery type was, itself, predictive of postoperative nutritional deficiency.

**Table 4:** Summary of individual logistic regression models

Nutrient	Odds ratio	95% CI	*p* value
Iron binding (*n *= 315)			
Preoperative nutritional deficiency	13.12	(5.88, 29.25)	< 0.001
Time			
12 months	5.93	(2.57, 13.68)	< 0.001
18 months	8.46	(2.93, 24.44)	< 0.001
Ferritin (*n *= 373)			
Preoperative nutritional deficiency	19.30	(10.85, 34.33)	< 0.001
Procedure type (reference: sleeve)			
Roux-en-Y	1.90	(1.09, 3.33)	0.024
Time (reference: 3 months)			
12 months	2.22	(1.41, 3.51)	0.001
18 months	2.46	(1.30, 4.68)	0.006
Zinc (*n *= 346)			
Preoperative nutritional deficiency	9.81	(3.50, 27.51)	< 0.001
Time (reference: 3 months)			
6 months	2.02	(1.03, 3.95)	0.040
Folate (*n *= 332)			
Preoperative nutritional deficiency	10.75	(1.81, 63.84)	0.009
Vitamin D250H (*n *= 372)			
Preoperative nutritional deficiency	10.17	(5.90, 17.50)	< 0.001
Time (reference: 3 months)			
9 months	3.50	(1.60, 7.66)	0.002
Vitamin B12 (n = 374)			
Preoperative nutritional deficiency	29.76	(2.75, 322.41)	0.005
Vitamin B1 (*n *= 320)			
Preoperative nutritional deficiency	2.78	(1.52, 5.10)	0.001

Each model was adjusted for preoperative deficiency, procedure type, and time at 3, 6, 9, 12, 18, and 24 months. Only statistically significant results are listed

## Discussion

Laboratory testing is expensive. Reducing unnecessary lab tests is a potential mechanism for decreasing unnecessary and low-value expenditures in a healthcare environment that is focused on value-based care and cost containment. Our findings show that having one or more preoperative nutritional deficiencies is associated with an increased risk of nutritional deficiencies post bariatric surgery, consistent with prior research supporting the rule, “Preoperative deficiencies are prone to postoperative deficiency” [14]. The initial step in determining the benefit and necessity of laboratory testing is an examination of lab results as per the recommended frequency/gold standard. Nationally there is an emphasis on value-based care, making it imperative that we try to identify which patients are at highest risk for nutritional abnormalities. This information would provide the basis to stratify the frequency of postoperative laboratory testing based on relevant risk factors, potentially adjusting current guidelines for nutritional testing post-bariatric procedures. The cost savings from decreasing the frequency of lab tests can be substantial and should be carefully examined if indeed lab results are providing limited actionable information.

Cost data in health care are largely variable and opaque. Understanding that reimbursement will vary and be less than total charges, the expense for laboratory testing following bariatric surgery is still substantial even if only a fraction of the total charge is realized at four time points for the initial postoperative year. Given the prevalence of obesity in the USA and the growing utilization of bariatric surgery as a treatment option, eliminating even one time point in the first year of follow-up could potentially result in millions of dollars of savings to the heath care system over time for this high volume procedure.

Our cohort of patients showed no significant difference in nutritional deficiencies between sleeve gastrectomy and Roux-en-Y. This suggests that the low incidence of nutrient deficiency is not attributed to the operation but more likely should be attributed to pre-existing comorbidities and medical history, specifically renal disease and prior surgery, and possibly to compliance with diet and vitamin supplementation postoperatively. There is also the question of the clinical relevance of these abnormalities and the potential undue burden being placed on patients in terms of both time and cost if the current level of postoperative lab testing is truly redundant. The level of nutritional deficiency in patients was often just below the threshold level, which likely yields no overt clinical manifestations or relevance. Therefore, many patients are likely to undergo unnecessary laboratory testing, thus expending unnecessary time and resources that do not benefit them, likely resulting in time off work and potentially lost income.

There are limitations inherent to this type of data analysis. Retrospective analyses can only access available data that is consistently collected. Not all laboratory results are input into EHRs in a retrievable format (e.g., scanned images), which resulted in a smaller sample size and likely accounts for some of the missing data across the six postoperative time points. Further, this is a single center study, which can lack external validity, limiting the generalizability of study findings. Finally, some nutritional deficiencies take years to accrue, and the relatively short follow-up times may contribute to an erosion of generalizability. Future research should continue to focus on identifying patients at risk for postoperative nutritional deficiencies that are clinically relevant and to support appropriately defining a timeline for postoperative nutrition evaluation.
